# Proximal junctional kyphosis and failure risk around the thoracolumbar junction: Impact of anterior malalignment, pelvic retroversion, and upper instrumented level selection

**DOI:** 10.1016/j.bas.2026.106082

**Published:** 2026-05-01

**Authors:** Mauricio Hansen, Lluis Vila, Aleksander Leszczynski, Carlos Aleman, Frank Meyer, Caroline Deck, Sleiman Haddad, Susana Núñez-Pereira, Ferran Pellisé, Anika Pupak, Ibrahim Obeid, Louis Boissière, Ahmet Alanay, Frank Kleinstück, Markus Loibl, Javier Pizones, Yann Philippe Charles, the European Spine Study Group (ESSG)

**Affiliations:** a Service de Chirurgie du Rachis, Hôpitaux Universitaires de Strasbourg, 1 Avenue Molière, Strasbourg, France; b Université de Strasbourg, 4 Rue Blaise Pascal, Strasbourg, France; c Icube Laboratory, UMR 7357, CNRS, Université de Strasbourg, Strasbourg, France; d Spine Surgery Unit, Vall d'Hebron University Hospital, Barcelona, Spain; e Spine Research Unit, Vall d’Hebron Research Institute, Barcelona, Spain; f ELSAN, Polyclinique Jean Villar, Clinique du Dos, Bruges, France; g Comprehensive Spine Center, Acibadem Maslak Hospital, Istanbul, Turkey; h Department of Spine Surgery, Schulthess Klinik, Zürich, Switzerland; i Spine Surgery Unit, La Paz University Hospital, Madrid, Spain

**Keywords:** Proximal junctional kyphosis, Proximal junctional failure, Adult spinal deformity, Sagittal alignment, Pelvic retroversion, Finite element analysis

## Abstract

**Introduction:**

Proximal junctional kyphosis/failure (PJK/PJF) remains a frequent and severe complication following adult spinal deformity (ASD) surgery. While alignment risk factors are known, the specific mechanical influence of anterior malalignment and pelvic retroversion across different fusion levels remains poorly understood.

**Research question:**

How do postoperative anterior malalignment and pelvic retroversion influence PJK/PJF risk and biomechanical forces at the proximal junction based on upper instrumented vertebra (UIV) selection?

**Material and methods:**

We retrospectively analyzed 351 ASD patients fused to the pelvis, stratified by UIV: lower thoracic (LT, T9–T11; n = 206) or upper lumbar (UL, T12–L2; n = 145). Radiographic spinopelvic alignment was evaluated. Additionally, a validated finite element model (FEM) of T10–pelvis and L2–pelvis constructs simulated progressive anterior offsets and pelvic retroversion to quantify UIV endplate compressive and shear forces.

**Results:**

PJK/PJF incidence was comparable between groups (LT: 22.4%, UL: 22.2%). In both cohorts, PJK patients exhibited greater 6-week postoperative global sagittal malalignment and pelvic retroversion. LT failures were driven by higher SVA and thoracic kyphosis, whereas UL failures associated with increased segmental T10–L2 kyphosis. FEM showed LT constructs experienced predominant compressive forces scaling with anterior offset, while UL constructs experienced predominant posterior shear forces. Pelvic retroversion offered negligible mitigation against compression and limited shear reduction.

**Discussion and conclusion:**

UIV selection dictates the biomechanical failure mechanism, not the overall PJK/PJF risk. LT instrumentation exposes the proximal junction to compression, whereas the UL spine is susceptible to shear-driven failure. Pelvic retroversion cannot compensate for residual anterior malalignment. Therefore, UIV choice must account for regional alignment and predictable force vectors.

## Highlights

• UIV selection dictates the mechanical failure mechanism (compression vs. shear). (76 chars).
• Lower thoracic (T9-T11) UIVs are more prone to compression-driven failure. (73 chars).
• Upper lumbar (T12-L2) UIVs are susceptible to posterior shear-driven failure. (74 chars).
• Pelvic retroversion cannot compensate for residual anterior malalignment. (70 chars).

## 1 Introduction

Proximal Junctional Kyphosis (PJK) and Proximal Junctional Failure (PJF) are among the most common mechanical complications following Adult Spinal Deformity (ASD) surgery (Lee et al., 2023; Zhao et al., 2021). Reported incidences range from 20% to 46% for PJK and up to 35% for PJF, most frequently occurring within the first postoperative year (Zhao et al., 2021). These complications significantly impair health-related quality of life and, in the case of PJF, are often associated with reoperation (Mohanty et al.; Ha et al., 2019). Consequently, risk prediction and prevention have become central goals in ASD surgery (Lee et al., 2023; Zhao et al., 2021; Mohanty et al.; Ha et al., 2019).

PJK results from a multifactorial interplay of biological, alignment-related, and surgical factors (Lee et al., 2023; Nakarai et al., 2024). Patient-specific characteristics such as age, weight, frailty, reduced bone mineral density, and sarcopenia have been associated with increased risk (Nakarai et al., 2024; tong et al., 2022). Surgically, long instrumentation and pelvic fixation reduce the postoperative sagittal compensation possibilities and can increase stress at the proximal junction (Safaee et al., 2018). Certain proximal instrumentation configurations including hooks, sublaminar bands, transition rods, cemented augmentation or tricortical screws are intended to decrease the PJK/PJF risk (Safaee et al., 2018; Liu et al., 2025). Avoiding residual global and segmental sagittal malalignment that trigger compensatory mechanisms is essential for the biomechanical behavior (Yilgor et al., 2017; Ham et al., 2021). Quantitative restoration of thoracolumbar alignment and proper segmental distribution, including ideal spine shape, lumbar apex and thoracolumbar inflection point, is mandatory (Yilgor et al., 2017). Moreover, age-related alignment changes such as Thoracic Kyphosis (TK), Sagittal Vertical Axis (SVA) increase, and a decline in efficient pelvic compensation through proportionate retroversion further contributes to PJK/PJF development (Ha et al., 2019; Ham et al., 2021; Charles et al., 2022a).

The choice of Upper Instrumented Vertebra (UIV) is critical, especially in long fusions (Yilgor et al., 2017; Ham et al., 2021). Shorter constructs ending in the upper lumbar spine aim to preserve mobility and reduce surgical morbidity, but may expose the adjacent mobile segment below the thoracolumbar junction to greater mechanical stress (Safaee et al., 2018). Extending fusion to the lower thoracic spine might modify the risk for PJK/PJF, as the sagittal UIV orientation is kyphotic (Ha et al., 2019; Yilgor et al., 2017). The biomechanical behavior of UIV and adjacent segments differs between lordotic lumbar and kyphotic thoracic regions (Ye et al., 2023). Biomechanical musculoskeletal simulations using the *AnyBody* model demonstrated that sagittal alignment influences the magnitude and direction of shear and compressive forces at the UIV (Ignasiak et al., 2023).

We hypothesize that residual postoperative anterior malalignment, TK and pelvic retroversion, could influence junctional forces depending on UIV location above or below the thoracolumbar junction. Such variations might influence PJK/PJF development.

The primary objective was to evaluate the association between anterior malalignment, compensatory pelvic mechanisms and PJK/PJF according to UIV above or below the thoracolumbar junction in ASD patients fused to the pelvis. The secondary objective was to correlate clinical findings from a multicenter register data with a biomechanical Finite Element Model (FEM) that quantifies shear and compression forces according to UIV and different degrees of anterior malalignment and pelvic retroversion.

## 2 Methods

### 2.1 Study design

This was a retrospective cohort study using prospectively collected multicenter data from an adult spinal deformity (ASD) registry. The study was approved by the respective institutional review boards of all participating centers. All patients provided informed consent for inclusion in the registry and for the use of anonymized data for research purposes. The study was conducted and reported in accordance with the STROBE reporting checklist.

### 2.2 Study population

Selection criteria were applied to the registry for patients operated between 2010 and 2023 in six centers. Inclusion criteria were age ≥18 years, ASD requiring posterior fusion to the sacrum or pelvis and with a UIV located from T9 to L2, minimum 2-year follow-up, and availability of complete clinical and radiographic dataset. Exclusion criteria were revision surgery with prior fusion across the thoracolumbar junction, congenital scoliosis, neuromuscular or syndromic spinal deformities. Additionally, patients were routinely screened for osteopenia and osteoporosis preoperatively using dual-energy X-ray absorptiometry (DEXA) scans. Femoral neck T-scores were utilized as the clinical threshold for evaluation, as spinal measurements in this deformity cohort were considered less accurate. Patients with incomplete Patient-Reported Outcome Measures (PROMs) at the 2-year follow-up, as well as those requiring postoperative reoperations or revisions, were retained in the final analysis.

Baseline clinical variables were collected: age, Body Mass Index (BMI), Charlson Comorbidity Index and Frailty Index. Patient Reported Outcome Measures (PROMs) were analyzed using the Scoliosis Research Society-22 (SRS-22) score and the Oswestry Disability Index (ODI).

Surgical variables accounted mainly for instrumentation length, and patients were stratified into two groups according to the UIV: Lower Thoracic (LT, T9–T11) and Upper Lumbar (UL, T12–L2). Furthermore, implant density per instrumented levels, double rods, rod alloy, interbody fusion, Smith-Petersen Osteotomies (SPO) and Pedicle Subtraction Osteotomies (PSO) were documented.

### 2.3 Radiographic assessment

Full spine standing radiographs were obtained preoperatively and at 6 weeks, 6 months, 1 year and 2 years postoperatively, and measured using validated KEOPS software (SMAIO, Lyon, France) (Maillot et al., 2015). The major curve Cobb angle and coronal malalignment defined as C7 plumbline – Central Sacral Vertical Line (CSVL) offset were assessed in the coronal plane. Sagittal parameters included: SVA C7, Pelvic Incidence (PI), Pelvic Tilt (PT), Sacral Slope (SS), TK T1–T12, Kyphosis T10-L2, Lumbar Lordosis (LL) L1–S1 and L4-S1. Kyphosis is expressed as positive and lordosis as negative values. Relative Spinopelvic Alignment (RSA), Relative Pelvic Version (RPV), Relative Lumbar Lordosis (RLL), Relative Spinopelvic Alignment (RSA) and Lordosis Distribution Index RSA were assessed, and the Global Alignment and Proportion (GAP) scores were calculated for each patient (Yilgor et al., 2017; Maillot et al., 2015). PJK and PJF were defined according to established radiographic and clinical criteria (Glattes et al., 2005): PJK was diagnosed as a UIV and UIV+2 Proximal Junctional Angle (PJA) increase ≥10° compared with the immediate postoperative measurement and PJF was defined as a PJK-associated mechanical failure due to vertebral body fracture of UIV or UIV+1, screw pullout, or soft-tissue disruption.

### 2.4 Finite element analysis

**Fig. 1:**
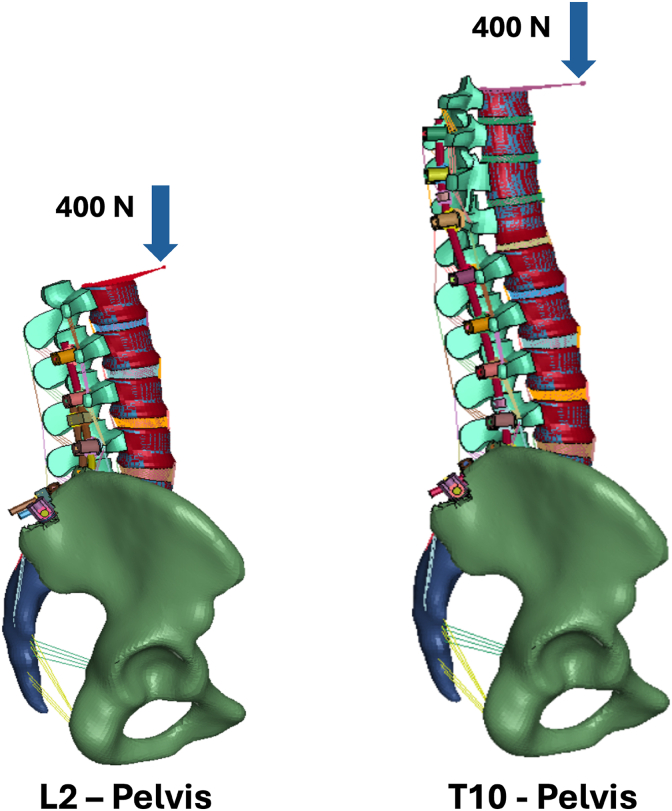
Finite Element Model with L2-pelvis (a), T10-pelvis (b) instrumentation, and offset load application.

**Fig. 2:**
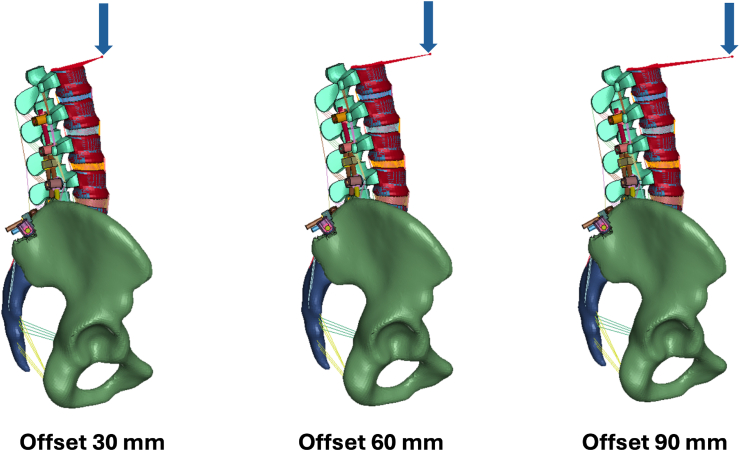
Three levels of anterior offset loading at 30 mm (simulating normal gravity line), 60 mm (mild malalignment) and 90 mm (severe malalignment).

**Fig. 3:**
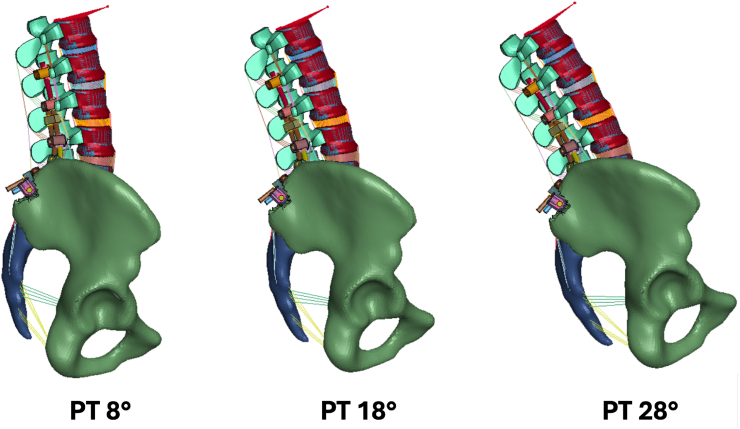
Three levels of pelvic retroversion with pelvic tilt of 8° (normal), 18° (mild retroversion) and 28° (severe retroversion).

A validated FEM of the thoracolumbar spine and pelvis was used to simulate the biomechanical effect of anterior malalignment and pelvic retroversion at the UIV. Material properties and boundary conditions when simulating standing posture and physiological load-displacement curves were defined according to published experimental data and previously described (Leszczynski et al., 2022). Two constructs were simulated: T10–pelvis and L2–pelvis, representing clinical LT and UL configurations (Fig. 1). The FEM was loaded with 400 N using a follower load. Simulations accounted for 3 levels of anterior offset loading (Fig. 2): 30 mm (simulating normal gravity line (Steffen et al., 2010)), 60 mm (mild malalignment) and 90 mm (severe malalignment). Three levels of pelvic retroversion were applied (Fig. 3): 8° (normal), 18° (mild retroversion) and 28° (severe retroversion). Each simulation quantified compressive and shear forces at the UIV endplate. The model was solved using ANSYS software (ANSYS Inc., Canonsburg, PA, USA).

### 2.5 Statistical analysis

Continuous variables were expressed as mean ± standard deviation (SD) and compared using Student's *t*-test or Mann–Whitney *U* test, as appropriate. Categorical variables were compared using chi-square or Fisher's exact test. Multivariate logistic regression was performed to identify independent predictors of PJK/PJF occurrence. A *p*-value <0.05 was considered statistically significant. All analyses were performed using SPSS Statistics (IBM Corp., Armonk, NY, USA).

## 3 Results

### 3.1 Demographic data

Among 2058 patients in the registry, 328 fulfilled inclusion criteria. Of these, 194 patients (59.1%) were assigned to the T9–T11 UIV group and 134 (40.9%) to the T12–L2 UIV group. The mean age of the overall cohort was 64.8 years (±10.2, Range 22-83), and 80.5% were female. The median follow-up was 4.9 years (±2.5, range 2–11.9 years). The average BMI was 26.5 (±4.5, Range 16-44.6), the Frailty Index 0.4 (±0.1, Range 0.2-0.7), and the Charlson Comorbidity Index 3.9 (±1.8, Range 0-11).

### 3.2 Baseline comparison of UIV groups

Baseline demographic variables and PROMs were comparable between UIV-UL (T12–L2) and UIV-LT (T9–T11) groups (Table 1), except that prior spine surgery was significantly (p = 0.003) more frequent in the UIV-UL group (64.2% vs 47.4%). The distribution of surgical parameters was similar in both groups (Table 2). Patients with longer instrumentation (UIV-LT group) had larger Cobb angles (38.7° vs 26.3°; p < 0.001), larger T10–L2 kyphosis (11.9° vs −1.7°; p < 0.001) and L4–S1 lordosis (−38.7° vs −34.3°; p < 0.001). (Table 3).

**Table 1:** Baseline Demographic and Clinical Characteristics Stratified by UIV Group. *Data are presented as mean ± standard deviation or n (%). UIV, Upper Instrumented Vertebra; BMI, Body Mass Index; ODI, Oswestry Disability Index; SRS-22, Scoliosis Research Society-22 patient questionnaire.*

Demographic & Clinical Data	UIV-UL Group (n = 134)	UIV-LT Group (n = 194)	p-value
Age (years)	64.7 ± 10.5	64.9 ± 10.1	0.892
Female, n (%)	101 (75.4)	163 (84.0)	0.052
BMI (kg/m^2^)	26.5 ± 4.5	26.5 ± 4.4	0.990
Charlson Comorbidity Index	4.0 ± 1.8	3.9 ± 1.8	0.404
Frailty Index	0.5 ± 0.1	0.4 ± 0.1	0.097
Etiology, n (%)			0.319
*Degenerative scoliosis*	91 (67.9)	120 (61.9)	
*Adult idiopathic scoliosis*	19 (14.2)	40 (20.6)	
*Other sagittal deformities*	24 (17.9)	34 (17.5)	
Prior Spine Surgery, n (%)	**86 (64.2)**	**92 (47.4)**	**0.003**
ODI (%)	51.9 ± 16.6	48.9 ± 16.8	0.117
SRS-22 Subtotal Score	2.6 ± 0.6	2.5 ± 0.6	0.940

**Table 2:** Surgical Variables Stratified by UIV Group and PJK/PJF Outcome. Data are presented as mean ± standard deviation or n (%). *PJK/PJF, Proximal Junctional Kyphosis/Failure; SPO, Smith-Petersen Osteotomy; PSO, Pedicle Subtraction Osteotomy.*

Variable	UIV-UL Group (n = 134)			UIV-LT Group (n = 194)		
	No PJK/PJF (n = 104)	PJK/PJF (n = 30)	p-value	No PJK/PJF (n = 151)	PJK/PJF (n = 43)	p-value
Instrumented Levels, n	6.9 ± 0.9	7.1 ± 1.0	0.195	9.9 ± 0.8	9.8 ± 0.6	0.314
Implant Density	1.8 ± 0.2	1.9 ± 0.1	0.055	1.8 ± 0.3	1.9 ± 0.2	0.727
Double Rod, n (%)	45 (43.3)	10 (33.3)	0.315	**58 (38.4)**	**7 (16.3)**	**0.007**
Rod Material, n (%)			0.14			0.527
*Cobalt Chrome*	41 (39.4)	16 (53.3)		70 (46.4)	18 (41.9)	
*Titanium*	45 (43.3)	11 (36.7)		69 (45.7)	21 (48.8)	
*Combination*	14 (13.5)	1 (3.3)		8 (5.3)	4 (9.3)	
*Stainless Steel*	0 (0.0)	1 (3.3)		1 (0.7)	0 (0.0)	
Interbody Fusion, n (%)	70 (67.3)	25 (83.3)	0.111	99 (65.6)	27 (62.8)	0.737
Number of Interbody Fusions	**1.2** ± **1.2**	**1.8** ± **1.2**	**0.033**	1.3 ± 1.3	1.3 ± 1.2	0.92
Osteotomy, n (%)	72 (69.2)	19 (63.3)	0.548	94 (62.3)	23 (53.5)	0.3
*SPO*	31 (29.8)	13 (43.3)	0.143	60 (39.7)	14 (32.6)	0.393
*PSO*	43 (41.3)	9 (30.0)	0.247	38 (25.2)	8 (18.6)	0.372

**Table 3:** Comparison of preoperative radiographic parameters between the Lower Thoracic (LT) and Upper Lumbar (UL) UIV groups. Data are presented as mean ± standard deviation. UIV, Upper Instrumented Vertebra; SVA, Sagittal Vertical Axis; RPV, Relative Pelvic Version; RLL, Relative Lumbar Lordosis; RSA, Relative Spinopelvic Alignment; LDI, Lordosis Distribution Index; GAP, Global Alignment and Proportion.

Preoperative Alignment	Lower Thoracic	Upper Lumbar	p-value
**Lumbosacral Fractional Curve**	14.6 ± 9.9	10.0 ± 8.1	**0.001 ∗**
**Major curve Cobb angle**	38.7 ± 19.2	26.3 ± 16.5	**<0.001 ∗**
**Coronal Balance (C7-CSVL)**	27.8 ± 27.0	22.4 ± 20.2	0.132
**Sagittal Balance (SVA)**	77.8 ± 68.8	75.5 ± 58.8	0.806
**Sagittal T2-T12**	33.6 ± 17.2	34.5 ± 16.5	0.604
**T1 Sagittal Tilt**	0.4 ± 6.2	0.8 ± 5.9	0.378
**Thoracolumbar L2-T10**	11.9 ± 18.0	−1.7 ± 15.1	**<0.001 ∗**
**Pelvic Incidence**	56.6 ± 13.4	57.1 ± 14.0	0.615
**Pelvic Tilt**	28.4 ± 10.1	27.1 ± 10.3	0.240
**Sacral Slope**	28.1 ± 10.6	30.0 ± 11.2	0.059
**L1-S1 Lordosis**	−31.5 ± 17.7	−32.2 ± 17.7	0.541
**Global Tilt**	36.8 ± 15.7	35.4 ± 14.0	0.694
**L4-S1 Lordosis**	−32.0 ± 13.7	−24.3 ± 13.0	**<0.001 ∗**
**RPV**	−14.2 ± 7.9	−12.7 ± 8.2	**0.035 ∗**
**RLL**	−32.6 ± 16.7	−32.2 ± 15.7	0.688
**RSA**	24.7 ± 13.6	23.0 ± 11.5	0.389
**LDI**	2.3 ± 7.5	2.2 ± 12.3	**<0.001 ∗**
**GAP Score**	9.6 ± 3.6	9.0 ± 3.3	**0.027 ∗**

### 3.3 Clinical PJK/PJF analysis

The incidence of PJK/PJF was comparable between groups (UIV-LT 22.4% vs UIV-UL 22.2%). In the global cohort, patients who developed PJK/PJF were older (67.2 vs 63.6 years; p = 0.003) and had significantly higher preoperative T10-L2 kyphosis (11.9° vs 4.8°; p = 0.003) No statistical differences were found for the rest of preoperative variables.

Table 2 demonstrates surgical parameters per UIV group. Patients had more interbody fusions in the UIV-UL group (p = 0.033) and the proportion of patients with double rods who developed PKJ/PJF was smaller in the UIV-LT group (p = 0.007).

Table 4 demonstrates pre- and postoperative radiographic alignment parameters per UIV group. Preoperatively, patients with PJK/PJF had a larger kyphosis T10-L2 (3.2° vs −3.1°; p = 0.045) in the UIV-UL group and (18.3° vs 10.2°; p = 0.010) in the UIV-LT group. Additionally, the SVA was larger in the UIV-LT group (101.0° vs 71.5°; p = 0.016).

**Table 4:** Radiographic Evolution Stratified by UIV Group and PJK/PJF Outcome. *Data are presented as mean ± standard deviation. UIV, Upper Instrumented Vertebra; UL, Upper Lumbar; LT, Lower Thoracic; PJK/PJF, Proximal Junctional Kyphosis/Failure; SVA, Sagittal Vertical Axis; RPV, Relative Pelvic Version; RLL, Relative Lumbar Lordosis; RSA, Relative Spinopelvic Alignment; LDI, Lordosis Distribution Index; GAP, Global Alignment and Proportion.*

Radiographic Parameter	UIV Group	Preoperative			6 Weeks Postoperatively			2 Years Postoperatively		
		No PJK/PJF	PJK/PJF	p-value	No PJK/PJF	PJK/PJF	p-value	No PJK/PJF	PJK/PJF	p-value
Major Curve Cobb Angle (°)	UIV-UL	26.2 ± 17.2	26.6 ± 14.1	0.911	21.4 ± 14.6	16.7 ± 11.6	0.108	21.3 ± 14.6	18.1 ± 12.0	0.293
	UIV-LT	38.8 ± 20.0	38.6 ± 16.1	0.972	25.4 ± 13.8	23.8 ± 9.0	0.493	24.1 ± 15.4	21.2 ± 10.5	0.249
Coronal Alignment (mm)	UIV-UL	22.9 ± 21.8	20.8 ± 13.8	0.627	17.6 ± 15.6	17.6 ± 14.3	0.999	18.6 ± 14.5	20.7 ± 17.7	0.539
	UIV-LT	28.3 ± 28.2	26.2 ± 22.2	0.653	19.4 ± 17.3	22.3 ± 17.5	0.336	16.7 ± 14.1	21.4 ± 17.7	0.081
SVA C7 (mm)	UIV-UL	75.8 ± 60.8	74.4 ± 52.4	0.912	41.5 ± 42.0	45.9 ± 40.3	0.616	47.1 ± 46.5	68.4 ± 49.3	0.047
	UIV-LT	71.5 ± 64.1	101.0 ± 80.8	0.016	27.1 ± 47.5	52.7 ± 69.8	0.006	37.1 ± 39.0	62.6 ± 42.7	<0.001
Pelvic Incidence (°)	UIV-UL	57.7 ± 14.1	55.2 ± 13.4	0.405	56.5 ± 13.0	54.1 ± 14.3	0.392	56.6 ± 16.4	57.1 ± 13.6	0.887
	UIV-LT	56.1 ± 12.9	58.1 ± 15.2	0.389	56.2 ± 12.7	57.9 ± 14.7	0.438	57.3 ± 12.3	57.7 ± 13.0	0.871
Pelvic Tilt (°)	UIV-UL	26.8 ± 10.2	28.1 ± 10.9	0.536	21.6 ± 8.9	23.8 ± 9.9	0.237	23.1 ± 10.7	27.1 ± 9.2	0.077
	UIV-LT	28.0 ± 9.7	30.0 ± 11.5	0.261	22.1 ± 8.5	26.2 ± 9.3	0.006	25.2 ± 8.0	28.4 ± 8.6	0.028
Sacral Slope (°)	UIV-UL	30.9 ± 11.0	27.1 ± 11.7	0.105	34.9 ± 10.4	30.3 ± 10.2	0.033	33.6 ± 11.0	30.0 ± 11.0	0.14
	UIV-LT	28.1 ± 10.2	28.2 ± 12.0	0.983	34.0 ± 10.0	31.7 ± 12.0	0.195	32.1 ± 9.5	29.3 ± 11.5	0.112
T1-T12 Kyphosis (°)	UIV-UL	35.5 ± 16.8	31.3 ± 15.4	0.229	43.1 ± 15.4	42.9 ± 15.2	0.935	42.3 ± 15.8	49.9 ± 15.6	0.035
	UIV-LT	33.2 ± 17.9	35.0 ± 14.4	0.543	48.1 ± 15.1	53.7 ± 15.5	0.035	50.3 ± 14.9	60.9 ± 17.1	<0.001
T10-L2 Kyphosis (°)	UIV-UL	−3.1 ± 15.3	3.2 ± 13.8	0.045	7.4 ± 10.4	15.6 ± 12.7	<0.001	9.1 ± 13.9	20.4 ± 13.0	<0.001
	UIV-LT	10.2 ± 18.2	18.3 ± 15.6	0.01	8.6 ± 13.0	17.6 ± 14.8	<0.001	11.8 ± 13.2	22.9 ± 13.0	<0.001
L1-S1 Lordosis (°)	UIV-UL	−33.7 ± 16.1	−27.0 ± 21.9	0.067	−48.0 ± 13.7	−43.1 ± 14.6	0.093	−46.3 ± 13.3	−43.7 ± 16.8	0.41
	UIV-LT	−31.2 ± 18.2	−32.5 ± 15.9	0.666	−52.4 ± 12.7	−52.6 ± 13.0	0.923	−50.5 ± 14.1	−50.1 ± 13.4	0.873
L4-S1 Lordosis (°)	UIV-UL	−23.8 ± 12.9	−25.9 ± 13.3	0.442	−35.4 ± 10.4	−32.8 ± 9.1	0.231	−33.9 ± 10.8	−32.8 ± 11.0	0.667
	UIV-LT	−31.7 ± 13.8	−33.1 ± 13.7	0.556	−36.2 ± 10.6	−39.5 ± 11.0	0.077	−36.0 ± 10.2	−36.4 ± 12.3	0.815
RPV (°)	UIV-UL	−12.1 ± 7.9	−14.5 ± 9.1	0.17	−7.4 ± 7.1	−10.6 ± 7.1	0.03	−8.8 ± 7.2	−12.7 ± 7.4	0.016
	UIV-LT	−14.0 ± 7.6	−15.1 ± 9.0	0.406	−8.1 ± 6.6	−11.5 ± 7.6	0.005	−10.7 ± 6.2	−13.7 ± 7.5	0.009
RLL (°)	UIV-UL	−31.0 ± 14.3	−36.2 ± 19.5	0.111	−16.0 ± 11.2	−19.4 ± 14.1	0.171	−17.8 ± 11.9	−20.7 ± 13.6	0.288
	UIV-LT	−32.6 ± 17.1	−32.5 ± 15.3	0.975	−11.4 ± 10.8	−12.3 ± 11.7	0.644	−14.0 ± 13.0	−14.6 ± 12.2	0.785
RSA (°)	UIV-UL	22.4 ± 10.9	25.0 ± 13.4	0.28	13.3 ± 8.5	17.2 ± 9.1	0.033	15.0 ± 9.3	21.9 ± 11.6	0.002
	UIV-LT	23.9 ± 13.6	27.5 ± 13.3	0.129	11.9 ± 9.0	17.5 ± 10.0	0.001	16.1 ± 8.7	22.4 ± 10.3	<0.001
LDI	UIV-UL	1.0 ± 1.3	6.2 ± 25.9	0.045	0.8 ± 0.3	0.9 ± 0.4	0.203	0.8 ± 0.4	0.9 ± 0.6	0.357
	UIV-LT	2.4 ± 7.9	2.1 ± 5.9	0.842	0.7 ± 0.2	0.8 ± 0.3	0.177	0.7 ± 0.2	0.7 ± 0.3	0.573
GAP Score	UIV-UL	8.9 ± 3.1	9.4 ± 3.8	0.462	5.9 ± 3.6	7.2 ± 4.3	0.096	6.7 ± 3.5	8.8 ± 3.4	0.007
	UIV-LT	9.4 ± 3.6	10.3 ± 3.3	0.151	5.0 ± 3.6	7.0 ± 3.6	0.001	6.0 ± 3.6	7.8 ± 3.4	0.006

At 6 weeks postoperatively, patients with PJK/PJF demonstrated some common patterns with greater residual pelvic retroversion and global sagittal malalignment in both UIV groups. When comparing PJK/PJF to non-PJK/PJF, the T10–L2 kyphosis was larger for UIV-UL (15.6° vs 7.4°; p < 0.001) and UIV-LT (17.6° vs 8.6°; p < 0.001). RPV was more negative for UIV-UL (−10.6° vs −7.4°; p = 0.030) and UIV-LT (−11.5° vs −8.1°; p = 0.005). RSA was larger for UIV-UL (17.2° vs 13.3°; p = 0.033) and UIV-LT (17.5° vs 10.0°; p = 0.001).

Postoperative differences existed for the UIV-LT group regarding TK and SVA, which were not observed in the UIV-UL group. UIV-LT patients had a larger T1-T12 kyphosis than UIV-UL at 6 weeks (53.7° vs 48.1°; p = 0.035), and this difference increased at 2-year follow-up (60.9° vs 50.3°; p < 0.001). Likewise, SVA was larger at 6 weeks (52.7° vs 27.1°; p = 0.006), and this difference increased at 2 years (62.6° vs 37.1°; p < 0.001).

Beyond radiographic parameters, Patient-Reported Outcome Measures (PROMs) at the 2-year follow-up demonstrated the functional impact of these mechanical complications across the entire cohort. Patients who experienced PJK/PJF exhibited a significantly lower SRS-22 subtotal score compared to those without complications (3.0 ± 0.8 vs. 3.3 ± 0.8, respectively; p = 0.003). While the mean Oswestry Disability Index (ODI) scores were worse in the PJK/PJF group (38.3% ± 18.0 vs. 31.7% ± 20.4), this difference did not reach statistical significance (p = 0.119).

Regarding bone quality, while the global incidence of PJK/PJF tended to be higher in patients with osteopenia or osteoporosis (31.1% vs. 20.5%; p = 0.087), this association reached statistical significance in the UIV-UL group. In this cohort, patients with poor bone quality had a PJK/PJF rate of 43.8% compared to 17.9% in those with normal bone density (p = 0.043). No such difference was observed in the UIV-LT group (26.7% vs. 22.7%; p = 0.685).

### 3.4 Finite element analysis

**Fig. 4:**
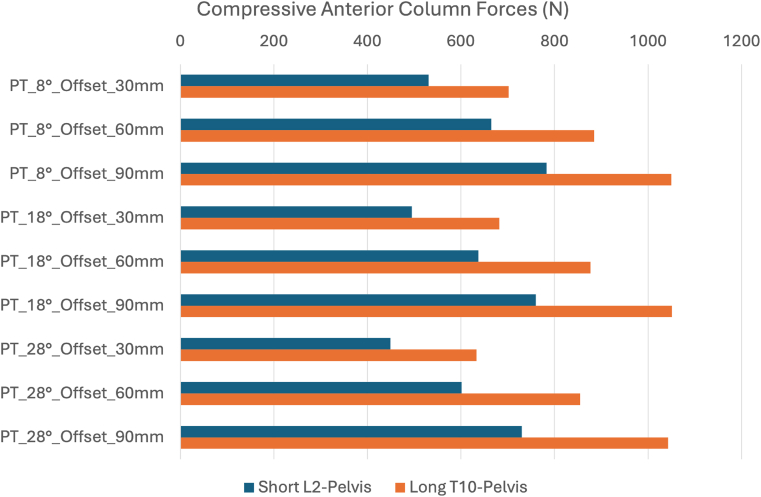
Compressive forces (N) at the UIV for 3 levels of anterior offset loading and pelvic tilt (PT) comparing L2-pelvis and T10-pelvis instrumentation.

Compressive forces at the UIV endplate were consistently higher in UIV-LT (T10–pelvis) configurations (Fig. 4). For both UIV groups, UIV endplate compression forces increased progressively as anterior offset distances (simulating anterior malalignment) for loading increased. Pelvic retroversion did not reduce compressive loading for any offset value.

**Fig. 5:**
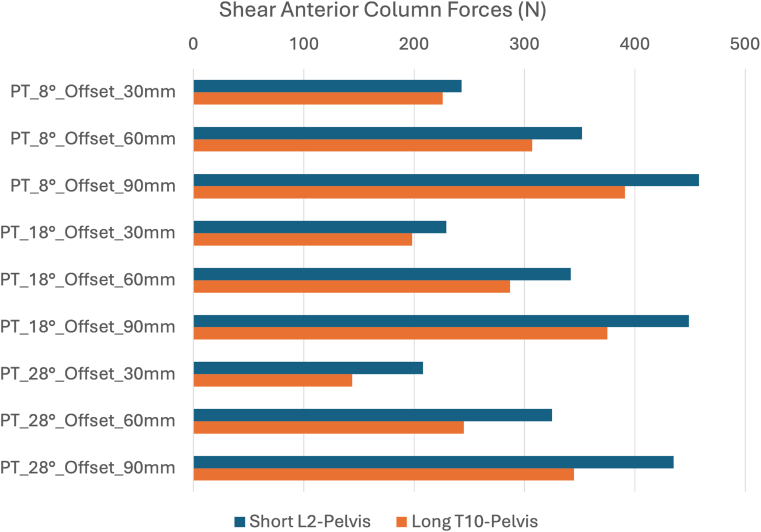
Shear forces (N) at the UIV for 3 levels of anterior offset loading and pelvic tilt (PT) comparing L2-pelvis and T10-pelvis instrumentation.

Shear forces were consistently higher in UIV-UL (L2–pelvis) configurations, with an anterior-posterior vector direction (Fig. 5). For both configurations, UIV endplate shear forces increased progressively as the anterior offset values increased. Pelvic retroversion had a decreasing (protective) effect on shear, but this effect was limited to 30 mm offset loading. At offsets of 60 mm and 90 mm, the influence of pelvic retroversion was lower.

In summary, the FEM predicted progressive increases in compression and shear forces with anterior malalignment and pelvic retroversion. The compressive component was predominant in the UIV-LT group, whereas the shear component was more important in the UIV-UL group.

## 4 Discussion

This combined clinical and biomechanical study identified distinct mechanisms of PJK/PJF associated with sagittal alignment following thoracolumbar versus lumbar fusions to the pelvis in patients with ASD. While the overall incidence of PJK/PJF was nearly identical between constructs with a UIV in the lower thoracic spine (T9–T11) and those with a UIV in the upper lumbar spine (T12–L2) (22.4% vs 22.2% respectively), the biomechanical patterns associated with failure differed between groups. (Hyun et al., 2017; Lu et al., 2025). It is also noteworthy that the UIV-UL group had a significantly higher rate of prior surgery (64.2% vs 47.4%; p = 0.003), which could be a potential confounding factor. This observation challenges the conventional view of PJK/PJF as a single uniform complication and suggests that the surgical decision of UIV placement does not merely alter the risk of failure, but fundamentally determines the mode of failure (Lee et al., 2023; Hyun et al., 2017). This finding, which aligns with incidence rates of 20%–40% reported in the literature (Zhao et al., 2021), does not simply reaffirm the high complication rate but rather deconstructs it, redirecting clinical focus from a singular emphasis on prevention to a more nuanced strategy of anticipating and mitigating specific, predictable mechanical stresses (Ashjaee et al., 2025).

The biomechanical basis of this clinical dichotomy was explored using FEM analysis. By simulating progressive anterior SVA shift and sagittal malalignment, the model demonstrated that constructs terminating at lower thoracic levels were subjected to consistently higher compressive forces, whereas those ending at upper lumbar levels experienced predominantly greater posterior shear forces. Accordingly, these distinct mechanical environments may predispose to different failure patterns, such as vertebral body fracture in compression-dominant settings and fixation failure or junctional subluxation in shear-dominant environments, consistent with the morphological subtypes described by Ha et al. (2019)

This might be partially explained by the UIV orientation, which is in a more lordotic position in the upper lumbar spine. The radiographic analysis has demonstrated that patients with preoperative T10-L2 kyphosis were prone to PJK/PJF. As UIV's at lower thoracic levels were located at the cranial end of this kyphotic segment, this might influence the orientation of the posterior shear vector. Therefore, the selection of the UIV level must be primarily guided by the preoperative sagittal profile of the thoracolumbar junction (TLJ). In cases where TLJ kyphosis is present preoperatively, extending the fusion to the thoracic spine is mandatory to prevent mechanical failure. Fusion termination at the upper lumbar levels should be considered only in patients without a pre-existing TLJ deformity. Additionally, bone quality strongly dictates UIV selection. The 43.8% failure rate in osteoporotic UIV-UL patients, compared to only 26.7% in the UIV-LT group, indicates that poor bone density is a relative contraindication for shorter constructs, favoring extension to the thoracic spine where stability is better maintained. Different biomechanical models were used to elucidate complex in vivo forces that are not directly measurable, thereby bridging the gap between clinical observation and mechanical etiology (Iyer et al., 2010; Oku et al., 2023; Ignasiak et al., 2018). This biomechanical premise was corroborated by the clinical analysis, which showed that in both UIV groups, the development of PJK/PJF was significantly associated with greater residual postoperative sagittal malalignment, measured by increased SVA, RSA, PT, and RPV. This confirms that sagittal malalignment acts as the trigger that initiates the cascade of destructive forces quantified by the FEM, a finding consistent with the literature identifying inadequate restoration of sagittal alignment as an important risk factor for PJK/PJF (Lafage et al., 2022). This may also help explain why alignment-based metrics such as the Global Alignment and Proportion (GAP) score have shown stronger predictive value for PJK in lower thoracic constructs, where compressive overload predominates, than in more cranial UIV configurations (Yilgor et al., 2017; Ye et al., 2023).

Ignasiak et al. (2023) combined clinical data and musculoskeletal *AnyBody* modeling, and confirmed that poorer postoperative alignment correlates with increased compressive and shear loads at the proximal junction between the instrumented and non-instrumented spine. However, this study did not specifically analyze UIV location above or below the thoracolumbar junction. Although shear forces at the proximal junction were reported, their regional direction was not a primary focus. In contrast, our FEM demonstrates posteriorly oriented shear forces at the caudal thoracic and thoracolumbar levels, likely related to differences in vertebral endplate orientation across the thoracolumbar junction (Ignasiak et al., 2023).

Furthermore, some specific aspects of the thoracolumbar junction need to be considered. This segment represents a critical transition zone between the rigid kyphotic thoracic spine and the mobile lordotic lumbar spine (Resnick et al., 1997; Prost et al., 2023; Ham et al., 2022). This mechanical transition likely explains why the thoracolumbar junction has consistently been identified as a high-risk region for PJK/PJF in clinical series (Lafage et al., 2024; Yasuda et al., 2017). Placing a UIV in the lower thoracic segment positions the instrumentation terminus where any anterior shift of the gravity line creates a long lever arm that exerts a dominant compressive force on the anterior column. This factor is present in ASD, but normal ageing, with SVA and TK increase, plays an additional role (Charles et al., 2022b). Our radiographic analysis showed that the TK and SVA component had an influence on PJK/PJF, mainly in the UIV-LT group, which aligns with predominant compressive forces in this group observed with the FEM. Conversely, a UIV in the upper lumbar spine is less influenced by TK and increased and compressive forces, as opposed to a UIV in the lower thoracic spine. Posterior shear seemed to be more important in the cranial LL.

A critical secondary finding is the limited efficacy of pelvic retroversion in ASD patients fused to the sacrum and pelvis as a primary compensatory mechanism. The FEM directly quantified these limitations. While pelvic retroversion demonstrated a modest capacity to decrease shear forces, this protective effect was confined to anterior offset loading at 30 mm, which reflects the normal trajectory of the gravity line as determined in healthy adults using radiographs and force platform measurements (Steffen et al., 2010). At greater malalignments, the influence of pelvic retroversion on shear forces decreased, and retroversion had a negligible effect on reducing compressive forces at any offset. These biomechanical data validate the clinical concept of pelvic rotation reserve previously described by Pizones et al. (2022) Our clinical findings support the FEM findings, as patients who developed PJK/PJF exhibited significantly greater residual pelvic retroversion postoperatively, leaving them in a state of active but failed compensation. This aligns with recent studies demonstrating that a high preoperative PT, signifying a depleted pelvic reserve, is an independent risk factor for PJK (Xu et al., 2025). Consequently, the primary surgical objective should not be merely to achieve an acceptable SVA that consumes the patient's reserve, but rather to achieve global spinopelvic realignment that restores this reserve, obviating the need for this biomechanically inefficient mechanism.

Several limitations must be acknowledged. The clinical analysis, while derived from a prospectively collected database, was retrospectively, introducing the potential for selection bias. The FEM, while robust, is inherently a simplification of the complex in vivo environment. It does not model the dynamic, stabilizing effects of active paraspinal musculature, which undoubtedly play a critical role. Furthermore, the model utilized idealized vertebral endplate orientations, which may not capture the full range of patient-specific anatomical variability. These constraints may reflect either the model's inherent simplifications or the multifactorial nature of PJK/PJF. In addition, while bone mineral density was evaluated, a quantitative assessment of paraspinal sarcopenia was not performed in our clinical cohort. Poor muscle mass and quality are known to compromise dynamic spinal stability, which could further reduce the tolerance of the proximal junction to compressive and shear loading (Nakarai et al., 2024; Eleswarapu et al., 2022; Pennington et al., 2025).

## 5 Conclusion

UIV selection in fusions to the pelvis for ASD modulates the type of mechanical stress at the proximal junction more than the overall rate of PJK/PJF. Global spinopelvic and segmental T10–L2 postoperative malalignment influences PJK-PJF in both UIV-LT and UIV-UL. Lower thoracic UIVs are more prone to compression-driven failure, linked to SVA and TK increase. Upper lumbar UIVs are more prone to posterior shear-driven failure, linked to the lordotic endplate position in the cranial LL. Therefore, the UIV choice should account for global and T10–L2 segmental thoracolumbar alignment and predictable force vectors.

## Funding

The ESSG received funding from Depuy-Synthes, Medtronic and Globus.

## Declaration of competing interest

The authors declare the following financial interests/personal relationships which may be considered as potential competing interests: F. Pellisé reports grants from J&J Medtech (Depuy), and consulting fees from Medtronic, Globus, Orthofix, and SpineArt. I. Obeid reports grants from Depuy and consulting fees from Spineart, Ethicon, and Clariance. A. Alanay reports consulting fees from Medtronic, Depuy, Globus, and Highridge. J. Pizones reports a research grant from Luis Álvarez (2024 IdiPaz and consulting fees from Stryker. F. Kleinstück, D. Jeszenszky, and T. Fekete report consulting fees or speaking agreements with Depuy Synthes Spine. Y.P. Charles reports consulting fees from Stryker and Clariance. F. Galbusera reports royalties from Elsevier Ltd. D. Haschtmann and T. Fekete report stock/stock options in Inno4Spine.

Additionally, the European Spine Study Group (ESSG) receives research grants and financial support from Depuy-Synthes, Medtronic, and Globus. The remaining authors (M. Hansen, L. Vila, A. Leszczynski, C. Aleman, F. Meyer, C. Deck, S. Haddad, S. Núñez-Pereira, A. Pupak, L. Boissière) declare that they have no known competing financial interests or personal relationships that could have appeared to influence the work reported in this paper.
